# The effects of transcranial direct current stimulation on gait in patients with Parkinson’s disease: a systematic review

**DOI:** 10.1186/s40035-021-00245-2

**Published:** 2021-06-29

**Authors:** Fateme Pol, Mohammad Ali Salehinejad, Hamzeh Baharlouei, Michael A. Nitsche

**Affiliations:** 1 Musculoskeletal Research Center, Isfahan University of Medical Sciences, Isfahan, Iran; 2 Department of Psychology and Neurosciences, Leibniz Research Centre for Working Environment and Human Factors, Dortmund, Germany; 3 Department of Neurology, University Medical Hospital Bergmannsheil, Bochum, Germany

**Keywords:** Transcranial direct current stimulation, Gait, Parkinson’s disease

## Abstract

**Background:**

Gait problems are an important symptom in Parkinson’s disease (PD), a progressive neurodegenerative disease. Transcranial direct current stimulation (tDCS) is a neuromodulatory intervention that can modulate cortical excitability of the gait-related regions. Despite an increasing number of gait-related tDCS studies in PD, the efficacy of this technique for improving gait has not been systematically investigated yet. Here, we aimed to systematically explore the effects of tDCS on gait in PD, based on available experimental studies.

**Methods:**

Using the PRISMA (Preferred Reporting Items for Systematic Reviews and Meta-Analyses) approach, PubMed, Web of Science, Scopus, and PEDro databases were searched for randomized clinical trials assessing the effect of tDCS on gait in patients with PD.

**Results:**

Eighteen studies were included in this systematic review. Overall, tDCS targeting the motor cortex and supplementary motor area bilaterally seems to be promising for gait rehabilitation in PD. Studies of tDCS targeting the dorosolateral prefrontal cortex or cerebellum showed more heterogeneous results. More studies are needed to systematically compare the efficacy of different tDCS protocols, including protocols applying tDCS alone and/or in combination with conventional gait rehabilitation treatment in PD.

**Conclusions:**

tDCS is a promising intervention approach to improving gait in PD. Anodal tDCS over the motor areas has shown a positive effect on gait, but stimulation of other areas is less promising. However, the heterogeneities of methods and results have made it difficult to draw firm conclusions. Therefore, systematic explorations of tDCS protocols are required to optimize the efficacy.

## Background

Parkinson’s disease (PD) is a progressive neurodegenerative disorder [1] caused by degeneration of the substantia nigra and dysfunction of the striatal pathway [2]. This leads to the increased GABAergic signaling from the output nuclei of the basal ganglia to the subcortical structures, including the thalamus. Consequently, the excitatory signaling from the thalamus to manifold cortical areas is decreased, leading to widespread cortical dysfunctions [3, 4]. Bradykinesia, dystonia, tremor, and postural balance disorders are prominent motor symptoms of PD [5]. Gait disturbances are debilitating impairments that increase the risk of falling in patients and negatively impact the quality of life [6]. Dopaminergic medication and deep brain stimulation are current standard interventions for PD [7, 8]. However, some motor symptoms do not respond well to medication and deep brain stimulation, and these treatments can also result in motor and sensory symptoms [7, 8]. Therefore, non-pharmacological, noninvasive therapies are currently increasingly being probed for their therapeutic value, including the non-invasive brain stimulation approaches.

Transcranial direct current stimulation (tDCS) is a non-invasive brain stimulation technique in which a weak electrical current is applied through the scalp. It alters cortical excitability by modulating the neuronal resting membrane potentials toward hyperpolarization or depolarization [9] and can produce acute and neuroplastic alterations of cortical excitability at the macroscale level of brain regions [10]. While anodal stimulation with standard protocols increases the cortical excitability, cathodal stimulation decreases it [11]. Stimulation for a few minutes produces neuroplastic after-effects, which share some characteristics with long-term potentiation and depression, including the involvement of glutamatergic synapses and calcium-dependency [12, 13]. Beyond these regional effects, tDCS modulates local intracortical circuits [13] and induces modifications of large-scale functional networks, which might also be useful for improving PD symptoms [14].

PD involves degeneration of dopaminergic neurons of the substantia nigra and impairment of dopaminergic circuits, especially motor circuits [15, 16]. Brain imaging studies with positron emission tomography and functional magnetic resonance imaging (MRI) have shown subcortical striato-nigral deficits in PD, which affect the activity of the cortical motor network [17]. In addition, the movement-related activity of the supplementary motor area (SMA) is significantly reduced in PD [17, 18]. It has been hypothesized that structural and functional connectivity between the SMA and the mesencephalic locomotor region, a region that contributes to the control of locomotion, is abnormal in PD [19, 20], and reduced activity of the SMA contributes to the pathogenesis of freezing of gait (FoG) [6]. Given the reduced activity of premotor and primary motor cortical regions in PD [21], there has been a growing interest in clinical application of tDCS to counterbalance respective alterations, and improve gait in PD.

Gait is a useful indicator for the therapeutic effects of motor rehabilitation in PD [22]. Anodal tDCS has the potential to enhance excitability and activity of motor regions in the brain and thus improve gait initiation. In animal models, tDCS even increased extracellular striatal dopamine levels [23], which might further ameliorate the motor symptoms of PD. Recent systematic reviews have confirmed that tDCS improves motor functions of PD patients [24, 25]. Moreover, some studies have suggested that tDCS combined with conventional gait rehabilitation therapy can have superior effects [26–28]. These effects might be partially due to the effects of tDCS on larger motor networks, given the dense connectivity of the motor cortex and the basal ganglia, and an impact of tDCS over the cortical regions to target the basal ganglia-thalamocortical motor circuits [26, 29–32]. In accordance, a functional MRI study has shown that the anodal tDCS over the primary motor cortex (M1) increases the functional connectivity between the left caudate nucleus and parietal association cortices and modulates the functional connectivity of cortico-striatal and thalamo-cortical circuits [33]. Furthermore, tDCS affects the functional connectivity between cortico-striatal and thalamo-cortical circuits [33], which is impaired in PD [34].

The efficacy of tDCS for gait improvement has not been reviewed specifically with respect to clinical effects and suitability of specific intervention protocols. In this systematic review, we set out to evaluate the effect of tDCS alone and in combination with other rehabilitation techniques on gait in PD patients, with consideration of specific parameters of tDCS that are assumed to affect the outcomes, such as the electrode position, stimulation intensity/duration, timing of medication, and performance. The main questions of this systemtic review are: 1) does tDCS improve gait parameters in PD patients? and 2) which protocols are best suited to improve gait in PD patients?

## Methods

### Data sources and search strategy

This systematic review was performed following the PRISMA (Preferred Reporting Items for Systematic Reviews and Meta-Analyses) guidelines [35] and was registered on 23 October, 2020, in the PROSPERO database (CRD42020177459).

We conducted an electronic search in the following databases: PubMed, Web of Science, Scopus, and PEDro, with the last search updated in February, 2021. The search terms were “Parkinson’s disease”, “Parkinsonian”, “transcranial direct current stimulation”, “gait”, “walking” and their respective synonyms (i.e., timed up and go, step, cadence, stride), and acronyms (i.e., tDCS).

### Study selection

Our research question was based on the PICOS (Population, Intervention, Comparison, Outcome measures, and study design) principle. Studies were included if the following inclusion criteria were met: (a) included sham-controlled tDCS; (b) employed patients with the diagnosis of PD or Parkinson syndromes; and (c) had a randomized controlled trial design (parallel groups or cross-over). Studies were excluded if they involved non-human subjects, written in a non-English language, or involved other techniques of transcranial stimulation (e.g., transcranial magnetic stimulation). The titles and abstracts of the retrieved papers were initially screened by two independent reviewers (HB and FP), and duplicates were eliminated by Endnote. After that, a full-text analysis was performed to determine whether these studies met the inclusion and exclusion criteria.

### Data extraction

After identifying relevant articles for inclusion in this study, data extraction was carried out independently by the two evaluators (HB, FP). The data included authors, year of publication, demographics of the participants, study design, tDCS protocol (electrode placement, stimulation intensity, duration, electrode size, and the number of sessions), combined treatments, outcome measures, main findings, and occurrence of adverse effects of tDCS. Disagreements between the evaluators were resolved through a third researcher (AS).

### Risk of bias

To assess the methodological quality of trials, the PEDro scale was used (http://www.pedro.org.au), which has been shown to represent high reliability and validity for this purpose [36, 37]. The PEDro scale includes 11 items that rate the internal and external validity of a study. The first item, which refers to external validity, is not considered as part of the final PEDro score [38, 39]. Two researchers (HB and FP) rated the articles independently, and any disagreements were resolved by discussion or, if required, with the consensus of a third reviewer (AS). The PEDro cut-points for determination of study quality were 9–10 (excellent), 6–8 (good), 4–5 (fair) and, below 4 (poor) [37].

## Results

### Data overview

**Fig. 1:**
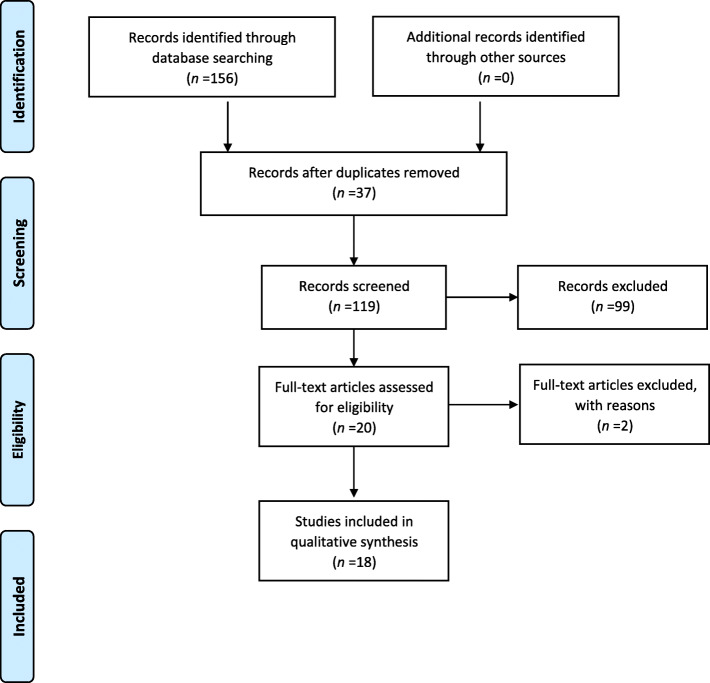
The PRISMA flow chart of included studies investigating the effects of transcranial direct current stimulation on gait symptoms in Parkinson's Disease

The initial search resulted in 156 articles. After eliminating 37 duplicates, 99 articles were excluded after screening by titles and abstracts. Two studies did not report sham stimulation results and were removed after reading the full text [40, 41]. Although our search space included not only PD but also Parkinsonian syndromes, all studies identified with a randomized design were conducted in PD. Eighteen articles, published between 2010 and 2021, were included in the final analysis of this study (Fig 1).

### Risk of bias

Thirteen studies had a PEDro score of 9 and 10, representing excellent quality [26, 32, 38, 39, 42–49], four studies had a score of 8 [50–53], and one study had a score of 7 [27]. Sixteen studies were designed double-blinded. Two studies were single-blinded but had otherwise good quality, as shown by a score of 8 [52, 53]. Three studies reported dropouts without conduction of an intention-to-treat analysis [27, 50, 51]. In all studies, the allocation procedure was not described (Table 1). Five studies delivered data showing that the participants could not discriminate sham from real tDCS [39, 44, 48–50], while the other studies did not monitor discrimination between real and sham stimulation by the participants. Inter-rater agreement with respect to the PEDro scale ratings was calculated using Cohen’s kappa coefficient, and the resulting *κ* value was 0.82.

**Table 1:** PEDro quality assessment of the included studies

Item	Benninger et al. 2010 [50]	Bueno et al. 2019 [42]	Chang et al. 2017 [43]	costa-Ribeiro et al. 2017 [26]	Criminger et al. 2018 [51]	da Silva et al. 2018 [27]	Dagan et al. 2018 [44]	Kaski et al. 2014 [54]	Lattari et al. 2016 [45]	Lu et al. 2018 [46]	Manenti et al. 2014 [47]	Mishra et al. 2021 [39]	Schabrun et al. 2016 [32]	Swank et al. 2016 [52]	Valentino et al. 2014 [53]	von Papen et al. 2014 [48]	Workman et al. 2020 [49]	Yotnuengnit et al. 2017 [38]
1	Y	Y	Y	Y	Y	Y	Y	Y	Y	Y	Y	Y	Y	Y	Y	Y	Y	Y
2	Y	Y	Y	Y	Y	Y	Y	Y	Y	Y	Y	Y	Y	Y	Y	Y	Y	Y
3	Y	Y	Y	Y	Y	Y	Y	Y	Y	Y	Y	Y	Y	Y	Y	Y	Y	Y
4	Y	Y	Y	Y	Y	Y	Y	Y	Y	Y	Y	Y	Y	Y	Y	Y	Y	Y
5	Y	Y	Y	Y	Y	Y	Y	Y	Y	Y	Y	Y	Y	Y	Y	Y	Y	Y
6	N	N	N	N	N	N	Y	N	N	N	N	N	N	N	N	N	N	N
7	Y	Y	Y	Y	Y	Y	Y	Y	Y	Y	Y	Y	Y	N	N	Y	Y	Y
8	Y	Y	Y	Y	Y	N	Y	Y	Y	Y	Y	Y	Y	Y	Y	Y	Y	Y
9	N	Y	Y	Y	N	N	Y	Y	Y	Y	Y	Y	Y	Y	Y	Y	Y	Y
10	Y	Y	Y	Y	Y	Y	Y	Y	Y	Y	Y	Y	Y	Y	Y	Y	Y	Y
11	Y	Y	Y	Y	Y	Y	Y	Y	Y	Y	Y	Y	Y	Y	Y	Y	Y	Y
total	8	9	9	9	8	7	10	9	9	9	9	9	9	8	8	9	9	9

1-Eligibility criteria were specified.

2-Subjects were randomly allocated to groups.

3-Allocation was concealed.

4-The groups were similar at baseline regarding the most important prognostic indicators.

5-There was blinding of all subjects.

6-There was blinding of all therapists who administered the therapy.

7-There was blinding of all assessors who measured at least one key outcome.

8-Measures of at least one key outcome were obtained from more than 85% of the subjects initially allocated to groups.

9-All subjects for whom outcome measures were available received the treatment or control condition as allocated or, where this was not the case, data for at least one key outcome was analyzed by “intention to treat”.

10-The results of between-group statistical comparisons are reported for at least one key outcome.

11-The study provides both point measures and measures of variability for at least one key outcome.

### Participant characteristics

A total of 322 individuals participated in the included studies. The mean age in each study ranged 62–72.4 years, and 173 of the participants were male (one study did not report the number of males and females [54]). The mean disease duration ranged 4.3–11 years among the studies (Table 2). The disease severity was quantified by the Hoehn-Yahr scale in 12 studies [27, 32, 38, 39, 42, 44, 45, 47, 49, 50, 52, 53] and the mean value reported in the studies was between 1.3 and 2.8.

**Table 2:** Characteristics of the included studies

Included Studies	Trial design	*n*	Age (years)	Sex (m, f)	Hoehn-Yahr	Mean duration of disease in years	Medication dosage (LED) in mg/day
Benninger et al. 2010 [50]	Parallel groups; double blind	25	63.9 (8.7)	16, 9	~ 2.8	~ 10	~ 1155
Bueno et al. 2019 [42]	Cross-over; double blind	20	64.45 (8.98)	12, 8	2.25 (0.63)	7.8 (5.32)	NR
Chang et al. 2017 [43]	Parallel groups; double blind	32	~ 63	20, 12	NR	9.3	~ 817
Costa-Ribeiro et al. 2017 [26]	Parallel groups; double blind	22	~ 62	15, 7	NR	~ 6.2	~ 815
Criminger et al. 2018 [51]	Cross-over; double blind	16	68.13 (9.76)	12, 4	NR	8.69 (9.7)	NR
da Silva et al. 2018 [27]	Parallel groups; double blind	21	66	10, 7	~ 2.5	~ 5.5	NR
Dagan et al. 2018 [44]	Cross-over; double blind	20	68.8 (6.8)	17, 3	2.5 (0.6)	NR	554.7 (401.1)
Kaski et al. 2014 [54]	Cross-over in two parallel groups; double blind	16	NR	NR	NR	NR	NR
Lattari et al. 2016 [45]	Cross-over; double blind	17	69.18 (9.98)	13, 4	2.35 (1.1)	7.06 (2.7)	748.29 (343.80)
Lu et al. 2018 [46]	Cross-over; double blind	10	66.3 (9.9)	7, 3	NR	NR	761.0 (362.2)
Manenti et al. 2014 [47]	Cross-over; double blind	10	67.1 (7.2)	6, 4	1.3 (1.1)	8.1 (3.5)	749.2 (445.5)
Mishra et al. 2021 [39]	Cross-over; double blind	20	63.9 (8.7)	14, 6	~ 2	NR	NR
Schabrun et al. 2016 [32]	Parallel groups; double blind	16	~ 67	10, 6	~ 2	~ 5.75	~ 626
Swank et al. 2016 [52]	Cross-over; single blind	10	68.7 (10.2)	8, 2	~ 2	7.9 (7.1)	NR
Valentino et al. 2014 [53]	Cross-over; single blind	10	72.3 (3.6)	5, 5	2.8 (0.5)	11 (4.9)	NR
von Papen et al. 2014 [48]	Cross-over; double blind	10	63 (9)	3, 7	NR	7 (6)	794 (360)
Workman et al. 2020 [49]	Cross-over; double blind	7	72.4 (6.4)	5, 2	1.9 (0.4)	4.3 (2.5)	889.8 (497.7)
Yotnuengnit et al. 2017 [38]	Parallel groups; double blind	60	65.0	33, 20	~ 2.5	~ 7.9	~ 863

*LED* Levodopa equivalent dosage; *NR* not reported. Mean (SD)

### The effect of tDCS on gait

#### The effect of motor area stimulation on gait

Ten studies assessed the effect of motor area stimulation on gait, and seven of them showed that tDCS improved gait. In the studies with positive findings, the anodal electrode was positioned 1–2 cm anterior to the vertex [26, 27, 50, 54], over the vertex [38], at the M1 corresponding to the leg with which the patient used to start walking after a freezing episode [53], or over the hotspot of the first dorsal interosseus muscle of the more affected side [48]. The stimulation intensity was 2 mA in six studies [26, 27, 38, 50, 53, 54]. Only one study, which tackled the motor cortex hand area, applied tDCS with 1-mA intensity [48]. The stimulation duration was 30 min in one study [38], 20 min in two studies [50, 53], 15 min in two studies [27, 54], 13 min in one study [26] and 10 min in one study [48]. Most of these studies included multiple-session interventions, comprising 10 sessions [26], 8 sessions [50], 6 sessions [38], and 5 sessions [53]. A single session approach was used in two studies [27, 54]. Six studies with positive findings measured gait immediately after the intervention, while in one study the first post-intervention assessment was performed one day after intervention [50]. The positive effects of tDCS lasted at least for three months in one study [50], for two months in one study [38], for one month in four studies [26, 38, 50, 53], and for two weeks in one study [53]. In two of these studies, tDCS was used as a stand-alone treatment [50, 53]. Other studies applied tDCS before gait training with visual cues [26], or before 1 Hz transcranial magnetic stimulation [48]. Other studies showed that tDCS had an impact on gait in PD patients who participated in a group-based exercise program [27], gait was improved in groups that received only real tDCS or a combination of real tDCS and physical therapy [38], or that only simultaneous tDCS and physical therapy improved gait in PD [54]. The state of medication during intervention was on in three studies [26, 27, 50]. The other four studies did not report the state of medication during intervention [38, 48, 53, 54]. Gait improvement was assessed *via* various methods, including the 10 m walk test [26, 50], timed up and go (TUG) test [26, 54], cadence [27, 38, 48], double support time [48], gait velocity [38, 54], stride length [48, 54], step length [38, 48], step width [38], 6-min walk test [54], stand walk sit test [53], and number of steps [48]. These outcomes were obtained during the medication-on state in two studies [27, 48]. In one study the gait was measured in both best medication-on state (considered by the patients and blinded rater to be the best response to their usual dopaminergic medication) and medication-off state (more than 12 h of withdrawal of dopaminergic medication) [50]. In another study outcomes were assessed in the off state and one hour after drug intake [26]. Two studies did not report the state of medication during assessment [38, 53] (Table 3).

**Table 3:** Intervention protocols and results in studies that targeted the motor area

Study	Polarity of target electrode	Electrode size (cm^2^)	Intensity (mA) Current density (mA/cm^2^)	Current density (mA/cm^2^)	Duration (min)	Number of sessions	Anatomical target (target electrode placement)	Target electrode placement	Return electrode placement	State of medication during intervention	Combined intervention	Online/offline tDCS	Adverse effects	Outcome measurements	Time points of assessment	State of medication during assessment	Results	Conclusion	Effect size (type)
Benninger et al. 2010 [50]	Anode	T = 97.5 R = 50 (two 25 cm^2^)	2 0.02	0.02	20	8 sessions (3 times per week)	M1, SMA (8 mm anterior to Cz or forehead above eyebrows)	8 mm anterior to Cz or forehead above eyebrows	Mastoid	On	–	Offline	Tingling	10-m walk test	Before, 24 h, 1 and 3 months after the last tDCS intervention session	On and off	Significant decrease of walking time in off-medication state	↑	NR
Costa-Ribeiro et al. 2017 [26]	Anode	T = R = 5 × 7	2 0.06	0.06	13	10 sessions (3 times per week)	M1, SMA (2 cm anterior to the Cz)	2 cm anterior to the Cz	Supraorbital area of the contralateral hemisphere of the more affected side	On	Gait training associated with cues	Offline	No	10-m walk test; TUG; cadence; stride length	Before, immediately and 1 month after intervention	On and off	10-m walk test and TUG in 1 month after intervention	↑	NR
da Silva et al. 2018 [27]	Anode	T = R = 5 × 7	2 0.06	0.06	15	1	M1, SMA (1.8 cm anterior to Cz)	1.8 cm anterior to Cz	Supraorbital area ipsilateral to the most affected side	On	Group-based exercise program	Offline	No	Gait kinematic analysis: stride length, cadence, duration, speed	Before, after	On	Gait cadence decreased	↑	0.87 (Cohen’s d)
Kaski et al. 2014 [54]	Anode	T = 10 × 4 R = 4 × 4	2 0.05	0.05	15	1	M1, SMA (10% to 20% anterior to Cz)	10% to 20% anterior to Cz	Inion	NR	Physical training focused on improving gait and balance or no combined intervention	Online	NR	6-min walk; TUG; gait velocity, stride length,	Before, after	NR	Gait velocity, stride length, TUG and 6-min walk test improved in the group that received both tDCS and physical training	↑	0.5 (Cohen’s d)
Lu et al. 2018 [46]	Anode	T = medium butterfly 2.0 cc, 8.1 cm^2^ R = 8.5 × 6	1 0.12	0.12	10	1	M1, SMA (1.8 cm anterior to Cz)	1.8 cm anterior to Cz	Centrally on the forehead	NR	–	Offline	No	Center of pressure movement and force onsets in gait initiation (FoG)	Before, immediately and every 12 min. For a total of 1 h after intervention	Off	No significant change	→	NR
Schabrun et al. 2016 [32]	Anode	T = R = 5 × 7	2 0.06	0.06	20	9 sessions (3 days per week)	M1 (C3)	Lt M1	Contralateral supraorbital	On	Dual task gait training with cues	Online	Tingling	Speed, step length, cadence, TUG	1 week before, 1 and 12 weeks after	On	No significant difference	→	NR
Valentino et al. 2014 [53]	Anode	T = R = 5 × 7	2 0.06	0.06	20	5 consecutive days	M1 (C4)	Right M1	Contralateral supraorbital	NR	–	Offline	No	Stand Walk Sit test (FoG)	Before, immediately after the 1st session, immediately, 2 days, 2 weeks and 4 weeks after 5th session	NR	Improvement in Stand Walk Sit test	↑	NR
von Papen et al. 2014 [48]	Anode and cathode	T = R = 5 × 7	1 0.03	0.03	10	1	M1 (Hotspot of first dorsal interosseus muscle)	Hotspot of first dorsal interosseus muscle	Contralateral frontal pole	NR	Transcranial magnetic stimulation with the frequency of 1 Hz	Offline	NR	Number of steps, step and stride length, cadence, double support time	Before, immediately and 30 min after stimulation	On	Improvement of number of steps, step and stride length, cadence, and double support time after anodal tDCS immediately and 30 min after stimulation	↑	NR
Yotnuengnit et al. 2018 [38]	Anode	T = R = 5 × 7	2 0.06	0.06	30	6 sessions (3 days per week)	M1 (Cz)	Cz	Supraorbital	NR	Physical therapy focused on improving gait or no combined intervention	Offline	Burning sensation	Walking speed, step length, step width, and cadence	Before, immediately, 2, 4, and 8 weeks after intervention	NR	Similar positive outcomes in all intervention groups lasted for 8 weeks	↑	NR

*T* target electrode, *R* reference electrode; *M1* primary motor cortex; *SMA* supplementary motor area; *NR* not reported; ↑: positive effect; →: no effect; *TUG* timed up and go; *NR* not reported; *FoG* freezing of gait; *tDCS* transcranial direct current stimulation

Different from those studies with positive findings, Dagan et al. [44] reported that tDCS over the motor cortex alone did not improve gait, but a single session of combined stimulation over the motor and the dorsolateral prefrontal cortex resulted in positive effects. However, this study differed from the above-mentioned studies with regard to the electrode size and type, and the stimulation intensity, which utilized relatively small electrodes and comparatively lower stimulation intensity (max, 1.5 mA). In the study by Schabrun et al. [32], tDCS at the left M1 applied for the first 20 min of each of the 9 dual task gait training sessions did not improve gait compared to the sham tDCS group. In this study, gait was not measured immediately after intervention and the first post-intervention assessment was performed one week after treatment. Gait was assessed under the dual task condition [32] (Table 3).

#### The effect of prefrontal area stimulation on gait

In eight studies, tDCS was applied over the dorsolateral prefrontal cortex (DLPFC) to improve gait [39, 42–45, 47, 51, 52]. In four studies that reported an improvement of gait by the intervention, the anodal electrode was placed over F3 or F4 [39, 43, 45, 47]. These studies assessed the effect of tDCS applied on five consecutive days [43] or in a single session [39, 45, 47] at intensity of 1 mA [43] or 2 mA [39, 45, 47]. The stimulation duration was 30 min [39], 20 min [43, 45] or 7 min [47]. While in three studies the participants received the intervention during the medication-on state [39, 43, 47], the other study did not report the state of medication during intervention [45]. In three studies, tDCS was used as a stand-alone treatment [39, 45, 47], while in one study the effects of isolated tDCS and a combination of tDCS and repetitive transcranial magnetic stimulation (rTMS) were compared [43]. The outcome measures included TUG [43, 45, 47], gait speed [39], FoG [43], and dynamic gait index [45]. The gait assessment was conducted in the medication-on phase in three studies [39, 43, 47], while the other study did not report the state of medication during assessment [45]. All the included studies targeting the DLPFC reported an immediate effect of tDCS. Furthermore, in the study by Mishra and Thrasher [39], gait was assessed during, as well as 15 min and 30 min after stimulation, and in another study the outcome measures were obtained one and five weeks after intervention [43]. Two studies did not report any follow-up assessment [45, 47] (Table 4).

**Table 4:** Intervention protocols and results in studies that targeted the DLPFC

Study	Polarity of target electrode	Electrode size (cm^2^)	Intensity (mA) Current density (mA/cm^2^)	Current density (mA/cm^2^)	Duration (min)	Number of sessions	Anatomical target (target electrode placement)	Target electrode placement	Return electrode placement	State of medication during intervention	Combined intervention	Online/offline tDCS	Adverse effects	Outcome measurements	Time points of assessment	State of medication during assessment	Results	Conclusion	Effect size (type)
Bueno et al. 2019 [42]	Anode	T = R = 5 × 7	2 0.06	0.06	20	1	DLPFC (F3)	F3	Fp2	NR	–	Offline	NR	TUG; Video gait analysis for time to cover a particular distance, gait speed, number of steps	Before, after	On	No improvement in TUG time and data from video gait analysis	→	NR
Chang et al. 2017 [43]	Anode	T = R = 5 × 5	1 0.04	0.04	20	5 consecutive days	DLPFC (F3)	F3	Contralateral supraorbital	On	Repetitive transcranial magnetic stimulation with 10 Hz frequency	Online	Headache	FoG questionnaire, modified Standing-Start 180° Turn Test (turning steps and turning time); TUG	Before, immediately and 1 week after 5 sessions of intervention	On	Significant improvement in FoG and ambulatory function in both groups. No significant difference in the between-group analyses	↑	NR
Criminger et al. 2018 [51]	Anode, cathode	T = R = 3 × 5	2 0.13	0.13	20	1	DLPFC (F3,F4)	F3, F4	F3, F4	On	Stationary bicycling; playing a video game of golf on Wii™	Online	Headache	TUG in ST and DT conditions	Before, after	On	No significant effect of tDCS on TUG	→	NR
Dagan et al. 2018 [44]	Anode	T = R = 3 (Pi-electrodes)	Max = 1.5 0.33	0.33	20	1	M1, DLPFC (AF4, CP1, F3, FC1, FC5, Cz)	AF4, CP1, F3, FC1, FC5, Cz	NR	NR	–	Offline	NR	FOG-provoking test; gait speed in 40 m walking, TUG	Before, after	NR	TUG, gait speed in 40 m and FoG improved by only multitarget stimulation	M1 alone: →; M1 and DLPFC: ↑	NR
Lattari et al. 2016 [45]	Anode	T = R = 5 × 7	2 0.06	0.06	20	1	DLPFC (F3)	F3	Contralateral supraorbital	NR	–	Offline	Tingling, itching	Dynamic Gait Index; TUG	before, after	NR	Improvement in dynamic gait index and TUG	↑	NR
Manenti et al. 2014 [47]	Anode	T = R = 5 × 7	2 0.06	0.06	7	1	DLPFC (F3 or F4)	F3 or F4	Contralateral supraorbital	On	–	Offline	No	TUG	Before, after	On	Decrease in TUG time comparing tDCS over F4 vs. sham tDCS	↑	NR
Mishra et al. 2021 [39]	Anode	T = R = 5 × 7	2 0.06	0.06	30	1	DLPFC (F3)	F3	Contralateral supraorbital	On	–	Online/offline	–	Speed	Before, during, immediately, 15 min, and 30 min after stimulation under ST and DT conditions	On	Improvement of gait under DT condition	↑	NR
Swank et al. 2016 [52]	Anode	NR	2	–	20	1	DLPFC (F3)	F3	F4	On	–	Offline	NR	TUG under three conditions: alone, with motor task, with cognitive task	Immediately after	On	No significant differences	→	0.07 to 0.45 *(Glass’ Δ)*

*T* target electrode, *R* reference electrode; *M1* primary motor cortex; *F3 *left DLPFC; *F4 *right DLPFC; *DLPFC* dorsolateral prefrontal cortex; *Fp2* right supraorbital area; *NR* not reported; ↑: positive effect; →: no effect; *TUG* timed up and go; *NR* not reported; *ST* single task; *DT* dual task; *FoG* freezing of gait; *tDCS* transcranial direct current stimulation

Dagan et al. [44] reported that the dual-site tDCS of the DLPFC and M1 improved the gait speed in PD. In that study, single-session tDCS was used as a stand-alone treatment, and the intensity and duration of stimulation were 1.5 mA and 20 min, respectively (Table 4).

In contrast, in the remaining three studies, DLPFC stimulation by anodal tDCS did not improve gait in PD. The intensity and duration of tDCS applied in these studies were 2 mA and 20 min respectively [42, 51, 52]. Criminger et al. paired tDCS with simultaneous stationary bicycling or a golf video game, and the stimulation intensity was 1 mA [51]. Swank et al. [52] assessed the single-session effect of isolated tDCS. The intensity and duration of tDCS were 2 mA and 20 min respectively. In contrast to the studies with positive findings [39, 43, 45, 47] that placed the return electrode over the contralateral supraorbital area, they used dual site stimulation in which both anodal and cathodal electrodes were placed over the DLPFC (Table 4).

#### The effect of tDCS over the cerebellum

Workman et al. [49] reported that the cerebellar tDCS at an intensity of 2 mA or 4 mA with a stimulation duration of 20 min did not improve gait in PD. However, 4-mA tDCS improved balance immediately after intervention, as assessed by the Berg balance scale (Table 5).

**Table 5:** Intervention protocols and results in the study that targeted the cerebellum

Study	Polarity of target electrode	Electrode size (cm^2^)	Intensity (mA) Current density (mA/cm^2^)	Current density (mA/cm^2^)	Duration (min)	Number of sessions	Anatomical target (electrode placement)	Target electrode placement	Return electrode placement	State of medication during intervention	Combined intervention	Online/offline tDCS	Adverse effects	Outcome measurements	Timepoints of assessment	State of medication during assessment	Results	Conclusion	Effect size (typr)
Workman et al. 2020 [49]	Anode	T = R = 5 × 7	2 and 4 0.06 and 0.11	0.06 and 0.11	20	1	Cerebellum (medial edge 1 cm below and 2 cm lateral to the inion over the cerebellar hemisphere contralateral to the more PD-affected side)	Medial edge 1 cm below and 2 cm lateral to the inion over the cerebellar hemisphere contralateral to the more PD-affected side	Upper arm or medial edge 1 cm below and 2 cm lateral to the inion over the cerebellar hemisphere ipsilateral to the more PD-affected side	NR	–	Offline	Burning sensation, itching, tingling, pins/needles	25 ft. walk test; TUG; 6-min walk test, Berg Balance Scale	Before, after	NR	No significant effects on gait parameters but improvement in Berg Balance Scale	→	NR

*T* target electrode; *R* reference electrode; *NR* not reported; →, no effect; *TUG* timed up and go; *tDCS* transcranial direct current stimulation

### The effect of tDCS on FoG

Four studies evaluated the effect of tDCS on FoG [43, 44, 46, 53]. In three studies, unilateral M1 stimulation [53], unilateral DLPFC stimulation [43] and M1 + DLPFC dual-site stimulation improved FoG in PD. The duration of intervention was 20 min in all the three studies and the intensity was 1 mA [43], 1.5 mA [44], or 2 mA [53]. In two of these studies, tDCS was applied as a stand-alone intervention [44, 53], while in one study it was combined with rTMS [43]. While two studies did not report the state of medication during intervention and assessment [44, 53], in the other study both intervention and assessment were conducted in the medication-on state [43]. The outcome measures were conducted immediately after intervention [43, 44, 53] and 2 days [53], 1 week [43], or 2 and 4 weeks [53] after intervention. In contrast, Lu et al. [46] reported no improvement of FoG by tDCS over the motor area. In that study, tDCS was applied as a stand-alone intervention at 1 mA intensity with 10 min duration, and FoG was however measured in the medication-off state immediately and up to 1 h after intervention.

### Reported side effects of tDCS

In six studies, participants reported no side effects [26, 27, 39, 46, 47, 53]. Seven studies reported mild and non-lasting side effects, including headache [43], tingling [32, 45, 49, 50], itching [45, 49], and burning sensations [38, 49]. Five studies reported no data about side effects [42, 44, 48, 52, 54].

## Discussion

To the best of our knowledge, this is the first systematic review to evaluate the efficacy of tDCS in the treatment of gait symptoms in patients with PD. In general, the study results showed that tDCS over motor areas holds some promise, whereas the prefrontal stimulation was comparatively less explored, and the results of available studies were heterogeneous. The results of different studies were however at least partially heterogeneous, which might be caused by the differences in intervention protocol, state of patients during intervention, and assessment, etc. The heterogeneity of outcome measures used, and relevant heterogeneities with respect to stimulation intensity, duration, electrode position, and other factors between studies, which were often intermingled, make it difficult or impossible to track back differences between studies to a single factor at present. Given these limitations of the available data set, we decided to conduct a narrative review.

A systematic review by Beretta et al. [24] has suggested that combined tDCS and motor intervention improves gait in PD. This review, however, did not include studies that used tDCS as a stand-alone treatment. Moreover, the results of the present review are in general accordance with systematic reviews suggesting the gait-improving effects of other non-invasive brain stimulation techniques for PD, and the positive effects of tDCS on gait in diseases other than PD. Nardone et al. [55] have suggested that rTMS targeting the M1 bilaterally decreases motor symptoms in PD, and a meta-analysis by Li et al. [56] has shown a significant effect of tDCS in improving mobility in individuals after stroke.

The present review suggests that placing the anode electrode anterior to the vertex is a promising approach to improving gait in PD. Stimulation of this area with relatively large electrodes may affect both M1 and SMA bilaterally. Since the cortical gait regions are represented bilaterally in the brain [57], bilateral stimulation may be required to modulate cortical excitability and improve gait. For the leg area of the primary motor cortex and the SMA, the more vertical orientation and deeper anatomical position as compared to the hand area of the motor cortex, make it more challenging to apply tDCS at the lower limb representations and SMA than at the upper limb representations [58]. However, transcranial magnetic stimulation showed that the anode tDCS anterior to the vertex can change the excitability of the leg motor and premotor regions, which suggests that tDCS over the leg motor and premotor regions can be used to improve locomotor control in PD patients [59].

In contrast to studies that showed a positive effect of motor area tDCS on gait in PD, Lu et al. [46] reported that bilateral anodal tDCS over the M1 did not improve FoG. In their study, tDCS was, however, applied only for 10 min at an intensity of 1 mA, and outcomes were measured during the medication-off state. Likewise, in two studies with unilateral rather than bilateral application of tDCS over the M1, no improvement of gait was reported [32, 44]. de Paz et al. [28] concluded in a systematic review that further research is required to identify the optimal stimulation targets for gait rehabilitation in neurological diseases.

There is some evidence supporting for an effect of tDCS over the DLPFC on gait in PD [39, 43–45, 47]. In one study, a significant improvement in FoG after 10 Hz rTMS over the DLPFC was reported [60]. The general rationale for this stimulation approach is that the frontal areas are relevant for locomotion [61]. Anatomical studies have shown that the DLPFC is an important part of the frontostriatal neural pathway that connects the frontal lobe regions with the striatum [62, 63]. In PD, the frontostriatal dysfunction is associated with significant deficits in executive functions, resulting in impaired walking [64]. A more recent study has shown higher DLPFC activity in PD patients compared to controls during both usual walking and walking while subtracting conditions, and impaired walking performance in PD patients as compared to controls only during the walking while subtracting task [65]. In addition, dual-site stimulation of M1 and DLPFC has exerted positive effects on gait in PD [44], which is consistent with the observation that FoG is caused by impaired communication among the prefrontal cortex, motor cortex, and subcortical structures [44, 66].

However, other studies targeting the DLPFC have not reported positive results [42, 51, 52]. In one study [51], tDCS was applied at a relatively low intensity (1 mA) and in combination with stationary bicycling or watching a golf video game, which are motor-related activities yet not as specific as the gait training exercise. In the study by Swank et al. [52], bipolar bilateral stimulation, in which both the anodal and the cathodal electrodes were placed over the DLPFC, might have resulted in the heterogeneous effects on this area because of the up- and downregulation of both hemispheres. In this connection, it is interesting to note that in the DLPFC studies with positive findings, a return electrode was positioned over the contralateral supraorbital area, rather than the DLPFC.

In the only study that applied tDCS over the bilateral cerebellum, the score of the Berg balance scale was increased, but no significant change of gait was found [49]. This result is not surprising, as the cerebellum plays a strong role in balance control [67]. These mixed findings indicate the importance of stimulation parameters (e.g., electrode placement, stimulation intensity, duration, repetition) that need to be adapted in order to improve treatment efficacy (e.g., [68]).

For the optimal electrode placement, in addition to anatomically defining areas, model-based optimization of electrode placement may be helpful, especially for targeting surface-away areas, such as the target regions for gait improvement. The individual models also allow for personalization of intervention, thereby improving the efficacy of the intervention and reducing the between-subject heterogeneity of results.

Based on available studies, the anode stimulation is a preferred target stimulation polarity. In this systemic review, all included studies have applied anodal tDCS, and two of them compared the effects of anodal and cathodal stimulation but reported no significant change of gait after cathodal tDCS [48, 51]. Since the activity of premotor and primary motor cortical regions is reduced in PD [21], the excitability-enhancing anodal tDCS may be suitable for restoring the activity of these areas [9].

In this review, most included studied applied tDCS at the intensity of 2 mA. tDCS at 1 mA intensity might be too low to facilitate the leg motor cortex and improve gait in PD [46], at least in some patients. This assumption is confirmed by the results of a study which showed that tDCS at 2 mA intensity performed better than 1-mA stimulation in modification of the postural response to an external perturbation in PD [69]. Furthermore, anodal tDCS with higher current density enhances the efficacy of tDCS to increase the excitability of the leg area of the motor cortex in healthy subjects [70]. Consistently, a study comparing the effects of anodal tDCS at different levels of intensity over the upper limb representations of M1 showed a trend of higher cortical excitability enhancements with increased current intensities [71, 72].

Regarding the stimulation duration, while most studies applied tDCS for 20 min, positive findings have also been reported for shorter stimulation durations. Nitsche and Paulus [73] have shown that 13 min of tDCS is sufficient to elevate the motor-evoked potential amplitudes at ~ 1 h after intervention in young healthy adults, and another study has reported no significant differences in the effects between 15 min and 30 min of anodal tDCS over the upper limb M1 on cortical excitability [71]. Although our review did not identify an association between the duration of stimulation and the efficacy in PD, studies in healthy humans have shown that longer stimulation durations can induce better effects [74, 75]. Therefore, a systematic evaluation of the impact of tDCS duration on its efficacy to treat PD symptoms would be required.

In the studies included in this review, both single-session [27, 44, 45, 47, 48, 54] and multiple-session intervention approaches [26, 38, 50, 53] improved gait in PD. Since the facilitatory effect of single-session tDCS on M1 excitability can last for about one hour after intervention [73], the single-session approaches might be well suited for screening of immediate intervention effects. In multiple-session approaches, the after-effects last for at least 3 months [50] in one study, 2 months [38] in one study, 1 month in three studies [38, 50, 53] and 2 weeks [38, 53] in two studies. One study has shown that the real and sham tDCS combined with visually cued gait training have similar positive effects immediately after the intervention, but at 1 month after intervention, these effects are only preserved in the real tDCS group [26]. The remote effects of tDCS on training-induced gait improvements may be due to the stabilization of training-induced plasticity, which could result in a long-lasting preservation of respective motor memories. The prolonged effects of repeated tDCS have also been reported for motor learning in healthy humans [76]. However, as the follow-up evaluations have not covered extensive durations after intervention, and comparisons of face-to-face interventions with different session numbers are missing, the exact duration of effects and the superiority of specific stimulation protocols remain to be determined. In addition, since the number of sessions in multi-session approaches is limited in the available studies, it might be the case that more extended interventions can cause larger, and more stable effects.

The results of the reviewed studies further suggest that combining tDCS with conventional gait training [26, 54], group-based exercise [27] or rTMS [48] may  enhance the effect of intervention. Kaski et al. [54] have reported superior effects of combined tDCS and physical training, as compared to the isolated tDCS or the isolated physical training, on gait rehabilitation in PD. Enhancing cortical excitability via tDCS in combination with other interventions might reinforce the positive effects of each, which can be translated into improved clinical outcomes, as compared with application of these interventions alone. The underlying mechanisms may be the synergistic induction of neuroplasticity, and enhanced motor network activation induced by these interventions and tDCS, as shown in healthy humans [77] and patients with motor deficits [78]. As mentioned above, this result mirrors those obtained in healthy humans by combining anodal motor cortex tDCS with motor learning [76]. In contrast, a systematic review by de Paz et al. [28] did provide conclusive results for an enhancing effect of adjunctive stimulation with current tDCS methods in combination with gait exercises in patients with neurological disorders.

With regard to the timing of combined intervention, it is so far unclear if tDCS during or before physical training is better suited in PD. Both online [54] and offline [26, 27, 48] tDCS resulted in positive effects in the included studies. It has, however, been shown that online stimulation has better effects than offline stimulation on motor learning in healthy humans [79]. Therefore, systematic face-to-face studies exploring the impact of online and offline stimulation on the effect of tDCS on gait in PD are needed to address this question.

In all studies that have reported the medication state during the intervention, tDCS was applied in the medication-on state. Dopamine modulates the motor cortex plasticity in M1. Hypo-dopaminergic states may prevent plasticity in PD patients and healthy humans [80, 81], thus it makes sense to conduct tDCS during the medication-on state. The state of medication is also important for the validity of the assessment of outcome measures. In six studies with positive findings, the participants were in the on state during assessment [27, 38, 45, 47, 48, 53]. However, Costa-Ribeiro et al. [26] found a positive effect of tDCS under both on and off conditions of medication. More investigations are needed to clarify whether tDCS can improve gait also in the off phase. The levodopa equivalent dosage was similar across the studies included in this review. Since dopaminergic medication can have non-linear dosage-dependent effects on plasticity in healthy humans [82, 83], it might be worthwile to assess the correlation between levodopa equivalent dosage and the intervention effects in future studies.

With respect to the demographic factors, the mean age of participants in the majority of the included studies was between 60 and 70 years, while only two studies reported a relatively higher mean age of > 70 years [49, 53]. For disease duration, the minima and maxima were 4.3 years [49] and 11 years [53], respectively. In the other studies, the mean disease duration was between 5 and 10 years. In 12 studies, the severity of the disease of the participants was evaluated by the Hoehn and Yahr scale, which had a mean value between 1.3 to 2.8 in these studies. This means that the patients were moderately affected, and patients with severe symptoms were under-represented. Although the present review showed no association between age, duration of disease, disease severity and gait improvement, future studies are needed to investigate the possible associations between these factors and the tDCS effects.

With respect to the impact of assessment methods on the outcome of interventions, most of the included studies used the TUG as the main outcome parameter, while others reported spatiotemporal parameters of gait. A systematic review by Mollinedo et al. [84] has shown good reliability and validity of TUG in PD [84] and also a high sensitivity of this test for monitoring medication-dependent effects [85]. Measurements of the spatiotemporal parameters of gait and balance can provide useful information on subtle effects that might not be identified by the TUG or other bed-side clinical tools. However, changes of these parameters may not provide information on clinically relevant effects for evey case. With respect to the reviewed studies, we did not find an association between the type of outcome assessment and the effect of tDCS, however, most of the studies differed in more than one intervention parameter. Therefore, the possible discernable effects of identical stimulation protocols on different outcome measures should be explored in future studies.

The loss of dopaminergic neurons in the substantia nigra pars compacta within the basal ganglia is a cause for gait impairments in PD [86]. In patients with PD, frontostriatal dysfunction has been associated with significant deficits in executive functions that are associated with difficulties in walking [64]. The cortical motor network and the movement-related activity of the SMA are altered in PD [17, 18], which contribute to the gait problems in PD [19, 20]. The lasting effect of tDCS on gait shows that although PD is a neurodegenerative disease, there is a potential for compensation by neuromodulation techniques.

### Limitations and suggestions

Besides the limitations already mentioned above, an important common limitation of the reviewed studies is the small sample size, making it difficult to generalize results. Future studies should include larger sample sizes. Furthermore, the substantial heterogeneity of study protocols makes it difficult to draw firm conclusions about the best suited protocols to improve clinical symptoms at present. Here, systematic evaluation of the impact of variations of specific aspects of tDCS protocols, which are known to affect the efficacy of the intervention, including the target area, the stimulation intensity, the session frequency, the number of sessions, set of combined vs stand-alone interventions, online vs offline stimulation in the case of combined interventions, association of stimulation effects with dopaminergic medication, is required to shape clinically useful interventions for future application. Additionally, most of the studies conducted so far have purely behavioral outcome parameters. For future studies, it will be relevant to add physiological assessments to clarify the mechanisms underlying the respective effects. The quality assessment of the included studies was conducted with PEDro, which evaluates the minimum requirements of study quality, e.g. not taking sample size into account as quality parameter. Most studies were proof of principle, and not designed to define clinically meaningful intervention protocols, which would require systematic evaluation of dosing and other intervention parameters. The effect size was only reported in a minority of studies and none of the papers delivered data about clinical meaningfulness of the effects. Such data are crucial for future clinical implementation, thus should be reported in future studies.

## Conclusions

The present review suggests that tDCS is a promising intervention approach to improving gait in PD. While applying anodal tDCS over the motor areas has shown a positive effect on gait in the majority of studies, stimulation over other areas like the DLPFC might be less promising. In addition, the small sample size and the heterogeneity of intervention protocols and outcome measures make it difficult to identify the best suited intervention protocols based on the current data, and to come to clear conclusions about the clinical usefulness of this intervention at present. Stimulation intensity, state of medication during intervention and assessment, and online versus offline tDCS in combination with traditional gait rehabilitation techniques are main aspects of variability of study protocols, which deserve further investigation. Although higher stimulation intensity has been shown to be more efficient in improving motor learning in healthy subjects, such a dosage-dependent effect needs to be tested directly in PD. The intervention has been conducted in the medication-on state in the studies as far as reported. This makes sense, because the plasticity-related effects of non-invasive brain stimulation are reduced in hypo-dopaminergic states; however, the impact of state of medication on the results of intervention needs to be explored directly. Furthermore, although the online tDCS has better effects than offline tDCS as shown for motor learning in healthy young adults, face-to-face studies in PD are lacking. The longest follow up was 3 months, but many studies only covered short timelines after intervention. More studies are thus needed to explore the duration of clinical effects of the intervention.

## Data Availability

Not applicable.

## Abbreviations

PD: Parkinson’s disease
tDCS: Transcranial direct current stimulation
SMA: Supplementary motor area
FoG: Freezing of gait
M1: Primary motor cortex
DLPFC: Dorsolateral prefrontal cortex
TUG: Timed up and go

## References

[1] Canning CG, Paul SS, Nieuwboer A. Prevention of falls in Parkinson's disease: a review of fall risk factors and the role of physical interventions. Neurodegener Dis Manag. 2014;4(3):203–21. 10.2217/nmt.14.22 25095816

[2] Jankovic J. Parkinson’s disease: clinical features and diagnosis. J Neurol Neurosurg Psychiatry. 2008;79(4):368–76. 10.1136/jnnp.2007.131045 18344392

[3] Takakusaki K, Tomita N, Yano M (2008). Substrates for normal gait and pathophysiology of gait disturbances with respect to the basal ganglia dysfunction. J Neurol.

[4] Takakusaki K (2017). Functional neuroanatomy for posture and gait control. J Mov Disord.

[5] Rizos A, Martinez-Martin P, Odin P, Antonini A, Kessel B, Kozul TK, Todorova A, Douiri A, Martin A, Stocchi F (2014). Characterizing motor and non-motor aspects of early-morning off periods in Parkinson's disease: an international multicenter study. Parkinsonism Relat Disord.

[6] Nutt JG, Bloem BR, Giladi N, Hallett M, Horak FB, Nieuwboer A (2011). Freezing of gait: moving forward on a mysterious clinical phenomenon. Lancet Neurol.

[7] Fox SH, Katzenschlager R, Lim SY, Ravina B, Seppi K, Coelho M, Poewe W, Rascol O, Goetz CG, Sampaio C (2011). The Movement Disorder Society evidence-based medicine review update: treatments for the motor symptoms of Parkinson's disease. Mov Disord.

[8] Baizabal-Carvallo JF, Jankovic J (2016). Movement disorders induced by deep brain stimulation. Parkinsonism Relat Disord.

[9] Nitsche M, Paulus W (2000). Excitability changes induced in the human motor cortex by weak transcranial direct current stimulation. J Physiol.

[10] Brunoni A, Nitsche M, Loo C. Transcranial Direct Current Stimulation in Neuropsychiatric Disorders*.* Springer; 2016.

[11] Stagg CJ, Nitsche MA (2011). Physiological basis of transcranial direct current stimulation. Neuroscientist.

[12] Nitsche M, Fricke K, Henschke U, Schlitterlau A, Liebetanz D, Lang N, Henning S, Tergau F, Paulus W (2003). Pharmacological modulation of cortical excitability shifts induced by transcranial direct current stimulation in humans. J Physiol.

[13] Nitsche MA, Seeber A, Frommann K, Klein CC, Rochford C, Nitsche MS, Fricke K, Liebetanz D, Lang N, Antal A (2005). Modulating parameters of excitability during and after transcranial direct current stimulation of the human motor cortex. J Physiol.

[14] Pereira JB, Junqué C, Bartrés-Faz D, Martí MJ, Sala-Llonch R, Compta Y, Falcón C, Vendrell P, Pascual-Leone A, Valls-Solé J, Tolosa E (2013). Modulation of verbal fluency networks by transcranial direct current stimulation (tDCS) in Parkinson's disease. Brain Stimul.

[15] Obeso JA, Marin C, Rodriguez-Oroz C, Blesa J, Benitez-Temiño B, Mena-Segovia J, Rodríguez M, Olanow CW (2008). The basal ganglia in Parkinson's disease: current concepts and unexplained observations. Ann Neurol.

[16] Wider C, Wszolek ZK (2008). Etiology and pathophysiology of frontotemporal dementia, Parkinson disease and Alzheimer disease: lessons from genetic studies. Neurodegener Dis.

[17] Sabatini U, Boulanouar K, Fabre N, Martin F, Carel C, Colonnese C, Bozzao L, Berry I, Montastruc J, Chollet F (2000). Cortical motor reorganization in akinetic patients with Parkinson's disease: a functional MRI study. Brain.

[18] Jahanshahi M, Jenkins IH, Brown RG, Marsden CD, Passingham RE, Brooks DJ (1995). Self-initiated versus externally triggered movements: I. An investigation using measurement of regional cerebral blood flow with PET and movement-related potentials in normal and Parkinson's disease subjects. Brain.

[19] Fling BW, Cohen RG, Mancini M, Nutt JG, Fair DA, Horak FB (2013). Asymmetric pedunculopontine network connectivity in parkinsonian patients with freezing of gait. Brain.

[20] Fling BW, Cohen RG, Mancini M, Carpenter SD, Fair DA, Nutt JG, Horak FB (2014). Functional reorganization of the locomotor network in Parkinson patients with freezing of gait. PLoS One.

[21] Playford E, Jenkins I, Passingham R, Nutt J, Frackowiak R, Brooks D (1992). Impaired mesial frontal and putamen activation in Parkinson's disease: a positron emission tomography study. Ann Neurol.

[22] Peppe A, Chiavalon C, Pasqualetti P, Crovato D, Caltagirone C (2007). Does gait analysis quantify motor rehabilitation efficacy in Parkinson's disease patients?. Gait Posture.

[23] Tanaka T, Takano Y, Tanaka S, Hironaka N, Kobayashi K, Hanakawa T, Watanabe K, Honda M (2013). Transcranial direct-current stimulation increases extracellular dopamine levels in the rat striatum. Front Syst Neurosci.

[24] Beretta VS, Conceição NR, Nóbrega-Sousa P, Orcioli-Silva D, Dantas LKBF, Gobbi LTB, Vitório R (2020). Transcranial direct current stimulation combined with physical or cognitive training in people with Parkinson’s disease: a systematic review. J Neuroengineering Rehab.

[25] Kim YW, Shin IS, Im Moon H, Lee SC, Yoon SY. Effects of non-invasive brain stimulation on freezing of gait in parkinsonism: a systematic review with meta-analysis. Parkinsonism Relat Disord. 2019;64:82–9. 10.1016/j.parkreldis.2019.02.029 30902526

[26] Costa-Ribeiro A, Maux A, Bosford T, Aoki Y, Castro R, Baltar A, Shirahige L, Moura Filho A, Nitsche MA, Monte-Silva K (2017). Transcranial direct current stimulation associated with gait training in Parkinson’s disease: a pilot randomized clinical trial. Dev Neurorehabil.

[27] da Silva DCL, Lemos T, de Sá FA, Horsczaruk CHR, Pedron CA, de Carvalho RE, de Oliveira LAS (2018). Effects of acute transcranial direct current stimulation on gait kinematics of individuals with Parkinson disease. To Geriatr Rehabi.

[28] de Paz RH, Serrano-Muñoz D, Pérez-Nombela S, Bravo-Esteban E, Avendaño-Coy J, Gómez-Soriano J (2019). Combining transcranial direct-current stimulation with gait training in patients with neurological disorders: a systematic review. J Neuroengineering Rehab.

[29] Schulz R, Gerloff C, Hummel FC (2013). Non-invasive brain stimulation in neurological diseases. Neuropharmacology.

[30] Ferrucci R, Mameli F, Ruggiero F, Priori A (2016). Transcranial direct current stimulation as treatment for Parkinson’s disease and other movement disorders. Basal Ganglia.

[31] Costa-Ribeiro A, Maux A, Bosford T, Tenório Y, Marques D, Carneiro M, Nitsche MA, Alberto Filho M, Monte-Silva K (2016). Dopamine-independent effects of combining transcranial direct current stimulation with cued gait training on cortical excitability and functional mobility in Parkinson's disease. J Rehabil Med.

[32] Schabrun SM, Lamont RM, Brauer SG. Transcranial direct current stimulation to enhance dual-task gait training in Parkinson's disease: a pilot RCT. PLoS One. 2016;11(6). 10.1371/journal.pone.0158497. 10.1371/journal.pone.0158497 PMC4928827 27359338

[33] Polanía R, Paulus W, Nitsche MA (2012). Modulating cortico-striatal and thalamo-cortical functional connectivity with transcranial direct current stimulation. Hum Brain Mapp.

[34] Dominey PF, Inui T (2009). Cortico-striatal function in sentence comprehension: insights from neurophysiology and modeling. Cortex.

[35] Moher D, Liberati A, Tetzlaff J, Altman DG (2009). Preferred reporting items for systematic reviews and meta-analyses: the PRISMA statement. Ann Intern Med.

[36] Maher CG, Sherrington C, Herbert RD, Moseley AM, Elkins M (2003). Reliability of the PEDro scale for rating quality of randomized controlled trials. Phys Ther.

[37] de Morton NA (2009). The PEDro scale is a valid measure of the methodological quality of clinical trials: a demographic study. Aust J Physiother.

[38] Yotnuengnit P, Bhidayasiri R, Donkhan R, Chaluaysrimuang J, Piravej K (2018). Effects of transcranial direct current stimulation plus physical therapy on gait in patients with Parkinson disease: a randomized controlled trial. Am J Phys Med Rehabil.

[39] Mishra RK, Thrasher AT (2021). Transcranial direct current stimulation of dorsolateral prefrontal cortex improves dual-task gait performance in patients with Parkinson’s disease: a double blind, sham-controlled study. Gait Posture.

[40] Hadoush H, Al-Jarrah M, Khalil H, Al-Sharman A, Al-Ghazawi S (2018). Bilateral anodal transcranial direct current stimulation effect on balance and fearing of fall in patient with Parkinson's disease. Neurorehabilitation.

[41] Ricci M, Di Lazzaro G, Pisani A, Scalise S, Alwardat M, Salimei C, Giannini F, Saggio G (2019). Wearable electronics assess the effectiveness of transcranial direct current stimulation on balance and gait in Parkinson’s disease patients. Sensors.

[42] Bueno MEB, do Nascimento Neto LI, Terra MB, Barboza NM, Okano AH, Smaili SM. Effectiveness of acute transcranial direct current stimulation on non-motor and motor symptoms in Parkinson's disease. Neurosci Lett. 2019;696:46–51. 10.1016/j.neulet.2018.12.017 30553865

[43] Chang WH, Kim MS, Park E, Cho JW, Youn J, Kim YK, Kim YH (2017). Effect of dual-mode and dual-site noninvasive brain stimulation on freezing of gait in patients with Parkinson disease. Arch Phys Med Rehabil.

[44] Dagan M, Herman T, Harrison R, Zhou J, Giladi N, Ruffini G, Manor B, Hausdorff JM (2018). Multitarget transcranial direct current stimulation for freezing of gait in Parkinson's disease. Mov Disord.

[45] Lattari E, Costa SS, Campos C, de Oliveira AJ, Machado S, Neto GAM (2016). Can transcranial direct current stimulation on the dorsolateral prefrontal cortex improves balance and functional mobility in Parkinson’s disease?. Neurosci Lett.

[46] Lu C, Amundsen Huffmaster SL, Tuite PJ, MacKinnon CD (2018). The effects of anodal tDCS over the supplementary motor area on gait initiation in Parkinson's disease with freezing of gait: a pilot study. J Neurol.

[47] Manenti R, Brambilla M, Rosini S, Orizio I, Ferrari C, Borroni B, Cotelli M (2014). Time up and go task performance improves after transcranial direct current stimulation in patient affected by Parkinson's disease. Neurosci Lett.

[48] Von Papen M, Fisse M, Sarfeld AS, Fink GR, Nowak DA (2014). The effects of 1 Hz rTMS preconditioned by tDCS on gait kinematics in Parkinson's disease. J Neural Transm.

[49] Workman CD, Fietsam AC, Uc EY, Rudroff T. Cerebellar transcranial direct current stimulation in people with parkinson’s disease: a pilot study. Brain Sci. 2020;10(2):96. 10.3390/brainsci10020096 PMC7071613 32053889

[50] Benninger DH, Lomarev M, Lopez G, Wassermann EM, Li X, Considine E, Hallett M (2010). Transcranial direct current stimulation for the treatment of Parkinson's disease. J Neurol Neurosurg Psychiatry.

[51] Criminger C, Swank C, Almutairi S, Mehta J (2018). Transcranial direct current stimulation plus concurrent activity may influence task prioritization during walking in people with Parkinson's disease - initial findings. J Parkinsonism Restless Legs Syndrome.

[52] Swank C, Mehta J, Criminger C (2016). Transcranial direct current stimulation lessens dual task cost in people with Parkinson’s disease. Neurosci Lett.

[53] Valentino F, Cosentino G, Brighina F, Pozzi NG, Sandrini G, Fierro B, Savettieri G, D'Amelio M, Pacchetti C (2014). Transcranial direct current stimulation for treatment of freezing of gait: a cross-over study. Mov Disord.

[54] Kaski D, Dominguez RO, Allum JH, Islam AF, Bronstein AM (2014). Combining physical training with transcranial direct current stimulation to improve gait in Parkinson's disease: a pilot randomized controlled study. Clin Rehabil.

[55] Nardone R, Versace V, Brigo F, Golaszewski S, Carnicelli L, Saltuari L, Trinka E, Sebastianelli L (2020). Transcranial magnetic stimulation and gait disturbances in Parkinson's disease: a systematic review. Neurophysiol Clin.

[56] Li Y, Fan J, Yang J, He C, Li S (2018). Effects of transcranial direct current stimulation on walking ability after stroke: a systematic review and meta-analysis. Restor Neurol Neurosci.

[57] Wennberg AMV, Savica R, Mielke MM (2017). Association between various brain pathologies and gait disturbance. Dement Geriatr Cogn Disord.

[58] Jeffery DT, Norton JA, Roy FD, Gorassini MA (2007). Effects of transcranial direct current stimulation on the excitability of the leg motor cortex. Exp Brain Res.

[59] Kaski D, Quadir S, Patel M, Yousif N, Bronstein AM (2012). Enhanced locomotor adaptation aftereffect in the “broken escalator” phenomenon using anodal tDCS. J Neurophysiol.

[60] Lee SY, Kim MS, Chang WH, Cho JW, Youn JY, Kim YH (2014). Effects of repetitive transcranial magnetic stimulation on freezing of gait in patients with parkinsonism. Restor Neurol Neurosci.

[61] Malouin F, Richards CL, Jackson PL, Dumas F, Doyon J (2003). Brain activations during motor imagery of locomotor-related tasks: a PET study. Hum Brain Mapp.

[62] Hone-Blanchet A, Edden RA, Fecteau S (2016). Online effects of transcranial direct current stimulation in real time on human prefrontal and striatal metabolites. Biol Psychiatry.

[63] Salehinejad MA, Ghanavati E, Rashid MHA, Nitsche MA. Hot and cold executive functions in the brain: A prefrontal-cingular network. Brain Neurosci Adv. 2021;5:23982128211007769. 10.1177/23982128211007769. 10.1177/23982128211007769 PMC8076773 33997292

[64] Maidan I, Nieuwhof F, Bernad-Elazari H, Reelick MF, Bloem BR, Giladi N, Deutsch JE, Hausdorff JM, Claassen JA, Mirelman A (2016). The role of the frontal lobe in complex walking among patients with Parkinson’s disease and healthy older adults: an fNIRS study. Neurorehabil Neural Repair.

[65] Ranchet M, Hoang I, Cheminon M, Derollepot R, Devos H, Perrey S, Luauté J, Danaila T, Paire-Ficout L (2020). Changes in prefrontal cortical activity during walking and cognitive functions among patients with Parkinson's disease. Front Neurol.

[66] Shine JM, Matar E, Ward PB, Frank MJ, Moustafa AA, Pearson M, Naismith SL, Lewis SJ (2013). Freezing of gait in Parkinson’s disease is associated with functional decoupling between the cognitive control network and the basal ganglia. Brain.

[67] França C, de Andrade DC, Teixeira MJ, Galhardoni R, Silva V, Barbosa ER, et al. Effects of cerebellar neuromodulation in movement disorders: a systematic review. Brain Stimul. 2018;11(2):249–60. 10.1016/j.brs.2017.11.015 29191439

[68] Salehinejad MA, Nejati V, Mosayebi-Samani M, Mohammadi A, Wischnewski M, Kuo MF, et al. Transcranial direct current stimulation in ADHD: a systematic review of efficacy, safety, and protocol-induced electrical field modeling results. Neurosci Bull. 2020;36(10):1191–212. 10.1007/s12264-020-00501-x PMC7532240 32418073

[69] Beretta VS, Vitório R, Nóbrega-Sousa P, Conceição NR, Orcioli-Silva D, Pereira MP, Gobbi LTB (2020). Effect of different intensities of transcranial direct current stimulation on postural response to external perturbation in patients with Parkinson's disease. Neurorehabil Neural Repair.

[70] Foerster Á, Dutta A, Kuo MF, Paulus W, Nitsche MA (2018). Effects of anodal transcranial direct current stimulation over lower limb primary motor cortex on motor learning in healthy individuals. Eur J Neurosci.

[71] Agboada D, Samani MM, Jamil A, Kuo MF, Nitsche MA. expanding the parameter space of anodal transcranial direct current stimulation of the primary motor cortex. Sci Rep. 2019;9:1–11. 10.1038/s41598-019-54621-0 PMC6890804 31796827

[72] Mosayebi-Samani M, Jamil A, Salvador R, Ruffini G, Haueisen J, Nitsche MA (2021). The impact of individual electrical fields and anatomical factors on the neurophysiological outcomes of tDCS: a TMS-MEP and MRI study. Brain Stimul.

[73] Nitsche MA, Paulus W (2001). Sustained excitability elevations induced by transcranial DC motor cortex stimulation in humans. Neurology.

[74] Monte-Silva K, Kuo MF, Hessenthaler S, Fresnoza S, Liebetanz D, Paulus W, et al. Induction of late LTP-like plasticity in the human motor cortex by repeated non-invasive brain stimulation. Brain Stimul. 2013;6(3):424–32. 10.1016/j.brs.2012.04.011 22695026

[75] Agboada D, Mosayebi-Samani M, Kuo MF, Nitsche MA (2020). Induction of long-term potentiation-like plasticity in the primary motor cortex with repeated anodal transcranial direct current stimulation - better effects with intensified protocols?. Brain Stimul.

[76] Reis J, Schambra HM, Cohen LG, Buch ER, Fritsch B, Zarahn E, et al. Noninvasive cortical stimulation enhances motor skill acquisition over multiple days through an effect on consolidation. Proc Natl Acad Sci U S A. 2009;106(5):1590–5. 10.1073/pnas.0805413106. 10.1073/pnas.0805413106 PMC2635787 19164589

[77] Polanía R, Nitsche MA, Paulus W (2011). Modulating functional connectivity patterns and topological functional organization of the human brain with transcranial direct current stimulation. Hum Brain Mapp.

[78] Allman C, Amadi U, Winkler AM, Wilkins L, Filippini N, Kischka U, Stagg CJ, Johansen-Berg H (2016). Ipsilesional anodal tDCS enhances the functional benefits of rehabilitation in patients after stroke. Sci Transl Med.

[79] Stagg C, Jayaram G, Pastor D, Kincses Z, Matthews P, Johansen-Berg H (2011). Polarity and timing-dependent effects of transcranial direct current stimulation in explicit motor learning. Neuropsychologia.

[80] Nitsche MA, Lampe C, Antal A, Liebetanz D, Lang N, Tergau F, Paulus W (2006). Dopaminergic modulation of long-lasting direct current-induced cortical excitability changes in the human motor cortex. Eur J Neurosci.

[81] Ueki Y, Mima T, Kotb MA, Sawada H, Saiki H, Ikeda A, Begum T, Reza F, Nagamine T, Fukuyama H (2006). Altered plasticity of the human motor cortex in Parkinson's disease. Ann Neurol.

[82] Fresnoza S, Stiksrud E, Klinker F, Liebetanz D, Paulus W, Kuo MF, Nitsche MA (2014). Dosage-dependent effect of dopamine D2 receptor activation on motor cortex plasticity in humans. J Neurosci.

[83] Monte-Silva K, Liebetanz D, Grundey J, Paulus W, Nitsche MA (2010). Dosage-dependent non-linear effect of L-dopa on human motor cortex plasticity. J Physiol.

[84] Mollinedo I, Ma Cancela J (2020). Evaluation of the psychometric properties and clinical applications of the timed up and go test in Parkinson disease: a systematic review. J Exerc Rehabil.

[85] Morris S, Morris ME, Iansek R (2001). Reliability of measurements obtained with the timed “up & go” test in people with Parkinson disease. Phys Ther.

[86] Muthukrishnan N, Abbas JJ, Shill HA, Krishnamurthi N (2019). Cueing paradigms to improve gait and posture in Parkinson's disease: a narrative review. Sensors (Basel).

